# Modern Treatment of Acute Internal Inflammations

**Published:** 1868-11

**Authors:** Wm. A. B. Norcom

**Affiliations:** Edenton, N. C.


### Modern Treatment of Acute Internal Inflammations.

BY WM. A. B. NORCOM, M. D., OF EDENTON, N. C.

Nothing so thoroughly arms a physician for his contest with disease, as a knowledge of its natural history, that he may be prepared to imitate and assist the curative changes nature so constantly strives to effect. Our remedies can only avail in so far as they aid natural operations.

In a lecture delivered by Sir Wm. Ferguson, at the Royal College of Surgeons of England, in June, 1865, I find the following: "The loss of confidence in much-vaunted remedies seems, in some respects, like a loss or diminution in our appliances—an abstraction from our powers, as it were. But in my opinion the correct view to take here is, that we are acquiring a knowledge of our own ignorance—that we are beginning to see that we have placed our faith erroneously. In short, that we have been taking honor to ourselves for that which has been justly due to nature. We begin to see the difference between blind empiricism and natural processes."

Says Anstie, on this point: "Without an observation of natural processes no medical man ever did great things for mankind, or for the advance of his art." * * * * "It was but yesterday that disease was universally regarded as something entirely foreign to the vital organism, which came to it from without, resided in it for a time, and then departed, exorcised by the physician's art. To-day we are inclined to take a less exalted view of our functions, to confess ourselves to be but the humblest assistants in those curative processes which Nature herself initiates, and very often carries through without our help, or even in spite of our ignorant interference. Together with such changed ideas, there must come a revolution in our modes of therapeutical inquiry; and notably, a disposition to compare those instances of the beneficial action of drugs which are well authenticated, with similar effects *produced by the unaided operation of natural causes.* And it is surely lawful to hope that even partial success in this direction, may prove more advantageous to the progress of our art, than the most brilliant reasoning which should presuppose the physician's power to effect radical alterations in the working of the vital agencies, whose operations we are only just beginning dimly and partially to understand."

And says Prof. P. Hughes Bennett: "If every young practitioner would dedicate his life to the careful elucidation of the natural progress of only one disease, he would do more for medical practice than has been accomplished by centuries of empirical trials of remedies."

And Dr. Todd, one of the brightest medical lights England ever produced, remarks thus: "Internal inflammation are cured, not by the ingesta administered, nor by the egesta promoted by the drugs of the physician, but by a natural process as distinct and definite as that process itself of abnormal nutrition to which we give the name of inflammation. Our interference either may aid, promote, and even accelerate this natural tendency to get well, or it may very seriously impair, and retard, and even altogether stop, that salutary process."

Prof. T. Gaillard Thomas, in a lecture to his class, in speaking of the natural history of disease, says: "Remain ignorant of it, and you shut the gates of the avenue which leads to progress in medicine; master it, and your therapeutic knowledge will become certain, and its application a science." He further says, in the same lecture, as the result of experience: "If fifty cases of pleurisy (the disease for which Sydenham prescribed so vigorously) be placed in bed, carefully nursed, dieted, guarded from deleterious influences, and receive not a particle of medicine of any kind, the probabilities are that not one case would end fatally; all would likely recover, unless some peculiarity of constitution, the unfavorable age of the person, or accidental complication should alter the result."

How very liable we are to be deceived in regard to the power and efficacy of drugs! Suppose in these fifty cases of pleurisy, some harmless medicine had been given! It might have been proclaimed as a specific. Or, suppose mercury, so frequently given by many physicians in this disease, had been administered to these patients, they may all still have recovered, but certainly not so quickly, nor so well as under the conditions mentioned by Dr. Thomas.

And, says the popular and accomplished professor of Physiology, Hygiene and General Pathology in the University of Maryland: "Now we substitute for the old compound prescriptions, simple remedies, given with a definite object, always recognizing the true position of nature as the curer, and medicine as her handmaid and assistant—laying great stress upon the observance of hygienic and sanitary laws."

Dr. Garrod tells us that he has seen many cases of severe rheumatic fever get rapidly well without the use of drugs, and that on simply colored or camphor water the improvement is often very quick and decided. In the Guy's Hospital Reports for 1865, are forty-one cases of rheumatic fever, thirty-seven treated by Dr. Gull, and four by Dr. G. O. Rees, "scarcely any medicine except mint water being given." Twenty-two were males, nineteen females; two only above the age of forty, the rest under thirty-five. The heart is mentioned as implicated in a large number of them. The average number of days from admission into hospital to comlete convalescence was, for the males, sixteen; females, twenty-one. The average duration of the acute symptoms in seven cases in which there was no evidence of the heart being involved, was eight days; in six cases in which the heart was decidedly affected, twenty-three days. From these cases what other inference can be drawn, except that mint water is a wonderful remedy for rheumatism, or that nature frequently triumphs over the disease? As mint water is known to be inert, we must accept the latter. Such facts as these should teach us a wholesome lesson. Suppose these cases had been salivated! Modern authors tell us that salivation neither shortens the duratlon of the disease, nor prevents cardiac complications. Then why practice it when, to say the least, the convalescence would necessarily be prolonged by the patient having to get well of the treatment as well as the disease? I mention this treatment particularly because I know that by many mercury is considered the "heroic" remedy in this as well as all other acute diseases.

By the foregoing remarks, I would not be understood to advise doing nothing for rheumatism; but we might learn the lesson to be careful in the use of powerful drugs, when the unaided powers of nature frequently effect a cure. In this disease, the alkaline treatment has proved highly successful.

A short time since, a lady of great intelligence told me that a few years ago she was treated for a pneumonia, the basis of which was *verat. viride* and low diet. Her medical attendant, a highly respectable physician, told her his object was to nauseate her to reduce her fever, which he very effectually did. She became cold, clammy, and almost pulseless. Active stimulation had to be resorted to, to save her from immediate death. She, however, finally recovered after a very tedious convalescence of six or eight weeks.

From an authentic source I heard of a case that occurred within a few years past, of bilious fever, which was terribly salivated. So offensive was the odor caused by the mortification consequent upon such ignorance and brutality, that one could not remain long in the room without sitting by a raised window. Yet this patient recovered, also, and was assured by the doctor that he would not have produced such a state of things, had it not been "necessary to save life."

Who can say that these remedies aided those patients to get well? A faithful report of the *convalescence* of such cases would be most interesting and instructive. Do not such recoveries, gentlemen, furnish a crowning demonstration of the great fact that Nature often triumphs over the doctor, and his treatment, too? What wonderful wisdom and goodness is displayed by the Almighty in permitting this to be so! If those only recovered who were properly treated, the inhabitants of this earth would grow less almost as rapidly as by a fiercely waged universal war. Call it what you may, the "*vis medicataix naturæ*," or, as Dr. Dickson says, life itself, there is a resistive force—an inherent curative power—that frequently thwarts, in the language of Dr. Thomas, "the machinations of misguided men."

Dr. Forget says that "Nature is stronger than physic and physicians; for if she were the slave of systems, the world would soon be a desert."

It is only by a careful and faithful study of her laws that we can hope to render to our patients that rational and effective aid, which it should always be the aim of the honest and conscientious votaries of our Heaven-born Art to give!

But let us pass on to alimentations. It has always seemed strange to me that nuritious food, so essential to maintain the organism in its integrity, should ever have been withheld in disease, at which time it is now proved to be so indispensable. When Nature is struggling to effect certain objects, which cannot be effected by art alone, and without which recovery could not occur, how very reasonable that we should give her the aid afforded by this agent! The system, worn and wearied by disease, and the blood impoverished, need support and repair; and food, suited to the powers of digestion and wants of the system is, above all others, the remedial means suggested alike by science and common sense. If the position sought to be established by Dr. Chambers be correct, there can be no question of the propriety of food at the very inception of disease. It is this: "That disease is, in all cases, not a *positive existence*, but a *negation;* not a *new access of action* but a deficiency; not a *manifestation of life*, but partial death; and therefore that the business of the physician is, directly or indirectly, not to *take away* material, but to add; not to *diminish function*, but to give it play; not to *weaken* life, but to renew life."

A New Hampshire paper says: "The greaest age ever attained in this State, by any person whose age was positively known, was that reached by Mr. Lovewell, of Dunstable, who died at 120. William Perkins, of Newmarket, reached 116; and Robert Macklin, of Wakefield, 115."

